# Differences in family functioning before and during the COVID-19 pandemic: an observational study in Peruvian families

**DOI:** 10.7717/peerj.16269

**Published:** 2023-12-08

**Authors:** Juan Carlos Bazo-Alvarez, David Villarreal-Zegarra, Wilder Iván Lázaro-Illatopa, Denisse Manrique-Millones, Miguel Ipanaqué-Zapata, María José Garcia, Oscar Bazo-Alvarez, Evelyn Goicochea-Ríos, Willy Valle-Salvatierra, Jackeline Edith García-Serna

**Affiliations:** 1 Escuela de Medicina, Universidad Cesar Vallejo, Trujillo, Peru; 2 Research Department of Primary Care and Population Health, University College London, London, United Kingdom; 3 Instituto Peruano de Orientación Psicológica, Lima, Peru; 4 Universidad Científica del Sur, Lima, Peru; 5 Universidad Privada Norbert Wiener, Lima, Peru; 6 PSYCOPERU Peruvian Research Institute of Educational and Social Psychology, Lima, Peru; 7 Universidad San Juan Bautista, Lima, Peru; 8 Universidad Católica Los Angeles de Chimbote, Chimbote, Peru

**Keywords:** COVID-19, Quarantine, Lockdown, Family functioning, Circumplex model

## Abstract

The COVID-19 pandemic has had a major impact on family relationships, as several families have lost family members due to COVID-19 pandemic and become physically and emotionally estranged due to lockdown measures and critically economic periods. Our study contrasted two hypotheses: (1) family functioning changed notably before and after the COVID-19 pandemic initiation in terms of cohesion, flexibility, communication and satisfaction; (2) balanced families have a greater capacity to strictly comply with quarantine (*i.e*., social confinement), compared to unbalanced families. We performed an observational study comparing family functioning between two independent groups, evaluated before and during the first wave of the COVID-19 pandemic in Peru. A total of 7,980 participants were included in the study. For the first hypothesis, we found that, during the pandemic, families became more balanced in terms of cohesion (adjusted before-during mean difference or β_1_ = 1.4; 95% CI [1.0–1.7]) and flexibility (β_2_ = 2.0; 95% CI [1.6–2.4]), and families were less disengaged (β_3_ = −1.9; 95% CI [−2.3 to −1.5]) and chaotic (β_4_ = −2.9; 95% CI [−3.3 to −2.4]). Regarding the second hypothesis, we confirmed that families with balanced cohesion (adjusted prevalence ratio or aPR = 1.16; 95% CI [1.12–1.19) and flexibility (aPR = 1.23; 95% CI [1.18–1.27]) allowed greater compliance with quarantine restrictions; while disengaged (aPR = 0.91; 95% CI [0.88–0.93]) and chaotic families (aPR = 0.89; 95% CI [0.87–0.92]) were more likely to partially comply or not comply with the quarantine. Finally, family communication (aPR = 1.17; 95% CI [1.11–1.24]) and satisfaction (aPR = 1.18; 95% CI [1.11–1.25]) also played a role in favouring quarantine compliance. This new evidence enlightens the family systems theory while informing future interventions for improving compliance with quarantine measures in the context of social confinement.

## Background

One of the biggest challenges that have affected people during the COVID-19 pandemic, and will continue to in its aftermath, is the degradation of mental health and wellbeing (Hossain et al., 2020; Pfefferbaum & North, 2020; Usher, Durkin & Bhullar, 2020b). The literature has documented increased uncertainty and anxiety in children, adolescents (de Figueiredo et al., 2021), and families during such an extremely stressful situation as the COVID-19 pandemic. The pandemic has forced families to live together 24 h a day, increasing the previous domestic tasks and roles but now with limited access to the outdoors (Hjálmsdóttir & Bjarnadóttir, 2021; Schieman et al., 2021). Families dealing with diverse activities such as remote work and home-schooling—due to the long-term closure of schools like in Peru—can succumb to the strain and lose family harmony and stability (Douglas et al., 2020). Many governments’ confinement and social distancing measures have reduced contact with extended family and friends, limiting the social support needed (Both et al., 2021). While technology and telecommunications have played an important role in maintaining social relationships outside the family of origin, there is evidence that more time spent using technology is associated with more stress and mental health problems (Park et al., 2023). Likewise, many families experienced financial stress due to job loss or income reduction, increasing the odds of health risk behaviour such as lack of sleep, unhealthy eating, smoking, and drinking (Sampson et al., 2021). When some family members are seriously sick or finally lose their lives, which is added to the scenario described above, stress at home can become very hard to handle and affect family functioning severely (Lebow, 2020).

According to the circumplex model (Olson, Waldvogel & Schlieff, 2019), family functioning is the interaction of emotional bonds among family members (cohesion) and the ability to adjust its structure in case to overcome evolutionary difficulties (flexibility). In the same model, family communication is a mediating factor that modifies family cohesion and flexibility throughout the family life cycle. A balanced family shows cohesion and flexibility levels that allow optimal functioning; it means that all its members can find the support and structure they need as individuals. Based on the idea of balanced families, the circumplex model comes with three main assumptions (Mirnics et al., 2010; Olson, Waldvogel & Schlieff, 2019). First, balanced families will generally function more adequately throughout the family life cycle than unbalanced families. Second, positive communication skills will allow balanced families to change their levels of cohesion and flexibility. Third, families will modify their cohesion and flexibility levels to cope with severe stressful situations (such as a pandemic). Our study focuses on exploring the third and first assumptions in the COVID-19 pandemic context.

Our first study hypothesis states that family functioning changed notably before and after the COVID-19 pandemic initiation in terms of cohesion, flexibility, communication and satisfaction. Some previous related investigations justify the evaluation of this hypothesis. Studies conducted during the COVID-19 pandemic have identified that stress coupled with enforcement measures (*i.e*., mandatory confinement) has increased the prevalence of family violence and mental health problems in families (Boserup, McKenney & Elkbuli, 2020; Chung et al., 2023; Usher et al., 2020a). In other health crisis contexts, other studies suggested that policies such as lockdowns, quarantines or closures can generate tensions within households, restricting rituals, norms, and family values, which, consequently, intensifies dysregulation of family functioning (DiGiovanni et al., 2004; Sprang & Silman, 2013). A more recent study assessed the effect of the COVID-19 pandemic on co-parenting quality and parents’ and children’s mental health, finding parents with increased depression symptoms and less co-parenting quality and children internalising/externalising more behaviour problems after the pandemic initiation (Feinberg et al., 2021).

Our second study hypothesis reads that balanced families have a greater capacity to strictly comply with quarantine (*i.e*., social confinement policies) than unbalanced families. Based on the first circumplex model assumption (Mirnics et al., 2010; Olson, Waldvogel & Schlieff, 2019), we anticipate that, in a pandemic context, balanced families have greater resources to take care of themselves and comply with quarantine policies than unbalanced families. This implies that families characterized by high levels of satisfaction, effective communication, strong cohesion, and adaptability are likely to exhibit a more favorable response to adhering to quarantine measures. Notably, families that maintain suitable levels of cohesion, flexibility, and communication tend to showcase the highest levels of overall family satisfaction (Olson, Waldvogel & Schlieff, 2019).

The majority of research concerning mental health in the context of the COVID-19 pandemic has been conducted within high-income countries (Boden et al., 2021; Kunzler et al., 2021). Nevertheless, it’s important to acknowledge that the greatest impact of the pandemic has been experienced by countries with low- to middle-income economies (LMICs). Notably, Peru has emerged as the nation with the highest mortality rate attributed to the COVID-19 pandemic (Reyes-Vega et al., 2021). In Peru, a state of emergency was declared on March 15, 2020, establishing specific sanitary measures such as quarantine and curfew to control the spread of contagion, in addition to the mandatory use of masks. Faced with this situation, the Peruvian Government provided economic aid (universal family bonus) to the low-income population to face the crisis. In addition, in the different regions we studied, they were exposed to differentiated measures to prevent COVID-19 pandemic, depending on the level of risk in each region, including traffic restrictions, reduction of public capacity and police surveillance to enforce the measures (Jaramillo Baanante & López Vargas, 2021).

## Methods

### Study design

We applied two different designs for studying each of the hypotheses proposed. For the first hypothesis, we performed an observational study comparing family functioning between two independents groups, evaluated before (January–March 2019) and during the first wave of the COVID-19 pandemic in Peru (June–December 2020). For the second hypothesis, we applied a cross-sectional design with the data collected during the COVID-19 pandemic (June–December 2020) to study the association between the family functioning and the compliance to the quarantine imposed by the Peruvian Government.

### Participants

One family member (participant) was surveyed to collect information about family functioning, quarantine compliance and other variables. Participants were either parents or kids in their own families. They conformed to a non-random sample that comes from 25 Peruvian cities. Data collection followed a mixed approach, including collection by pollsters and online surveys. The inclusion criteria were to be 16 to 99 years old and consent to participate in the study. The exclusion criterion was to have missing data on the family functioning data. The minimum sample size necessary for our analysis is 1,302, as determined for a multiple linear regression model. This calculation is based on a two-tailed distribution, a 0.05 margin of error, and a desired power level of 95%. We’ve factored in the presence of five covariates for adjustment, and we used G*Power version 3.1.9.7.

Data collection was carried out at two different times and in two different groups. The firsts sample was evaluated in the general population of the city of Chimbote in northern Peru (Department of Ancash) with an estimated population of about half a million. The selected city is an urban context, whose inhabitants have an average income, access to basic services, transportation, education, and health. The data collected was between January and February 2019 (before the COVID-19 pandemic). In 2019, at the national level, there were no health restrictions preventing people from moving. The evaluation strategy was going house by house inviting people to participate voluntarily in the study. During data collection, sociodemographic data, FACES IV, FSS, and FCS were extracted from all those evaluated.

The second sample was evaluated in the university students during the quarantine in different cities of Peru, between June and December 2020 (during the pandemic). The data collected in 2020 came from participants from different cities in Peru. We sought to evaluate a sample that was sufficiently heterogeneous to allow comparisons between different regions of Peru. Families were evaluated from northern Peru (Ancash, Cajamarca, La Libertad, Lambayeque, Piura, and Tumbes), southern Peru (Arequipa, Cuzco, Madre de Dios, Moquegua, Puno, and Tacna), central Peru (Apurimac, Ayacucho, Huancavelica, Huanuco, Ica, Junin, and Pasco), eastern Peru (Amazonas, Loreto, San Martin, and Ucayali), and Lima (Lima and Callao). Following the recommendation of social distance, participants were recruited from online advertisements, email, and social media in the second sample. During data collection were extracted: sociodemographic information, Family Adaptability and Cohesion Evaluation Scale (FACES IV), Family Communication Scale (FCS), and Family Satisfaction Scale (FSS).

### Variables for the first hypothesis

#### Exposure (first hypothesis)

The exposure variable for the first hypothesis was the initiation of the COVID-19 pandemic (Jan–Mar 2020). Those participants assessed in 2020 were considered exposed, and those assessed in 2019 were considered unexposed.

#### Outcome (first hypothesis)

Family functioning is the outcome, although it is a complex and multidimensional variable. We defined family functioning using the circumplex model of couple and family systems (Olson, 2011), which proposes family cohesion, flexibility, communication, and satisfaction as dimensions to define family functioning.

*Family cohesion and flexibility*. The Family Adaptability and Cohesion Evaluation Scale (FACES IV) assessed family cohesion and flexibility. It is a Likert-type scale designed by Olson, formed by six dimensions of seven elements each, with response options ranging from one (strongly disagree) to five (strongly agree). For interpretations, the six dimensions are grouped into two blocks, two balanced and four unbalanced dimensions (Olson, 2011). The balanced dimensions are balanced cohesion and balanced flexibility. The unbalanced dimensions are Disengaged, Enmeshed, Rigid and Chaotic. The circumplex model states that there are balanced or functional levels of cohesion (balanced cohesion) and extreme or dysfunctional levels of family cohesion that are linked to high levels of cohesion (Enmeshed) or low levels of cohesion (Disengaged). It is hypothesised that extremely high levels of flexibility (Chaotic) and extremely low levels of flexibility (Rigid) are problematic for the family system, while balanced levels are more functional (balanced flexibility). The scales had very good levels of reliability: disengaged = 0.87, enmeshed = 0.77, rigid = 0.83, chaotic = 0.85, balanced cohesion = 0.89, and balanced flexibility = 0.80 (Olson, 2011). For the Spanish version, the study showed adequate levels of reliability and validity, obtaining the following reliability coefficients: disengaged = 0.77, enmeshed = 0.63, rigid = 0.68, chaotic = 0.65, balanced cohesion = 0.71, and balanced flexibility = 0.64 (Costa Ball et al., 2009). Other psychometric studies in Peru are consistent with these results (Bazo-Alvarez et al., 2016; Villarreal-Zegarra & Paz-Jesús, 2017).

*Family communication*. Communication is a facilitating dimension, which means that good communication helps couples and families modify their cohesion and flexibility levels, helping to better face the demands of critical situations. Communication was evaluated with the Family Communication Scale (FCS). This is a Likert-type instrument with ten items and responses on ordinal scaling, ranging from one (strongly disagree) to five (strongly agree). Family communication is defined as the ability of the family system to transmit information, feelings, emotions, and needs among its members (Olson, Gorall & Tiesel, 2006). The original version presents adequate levels of reliability (α = 0.90) and validity (Olson, Gorall & Tiesel, 2006). Its adaptation in the Peruvian population was made in university students, presenting evidence of validity by internal structure (CFI > 0.95, RMSEA < 0.08) and optimal reliability (α > 0.85) (Copez-Lonzoy, Villarreal-Zegarra & Paz-Jesús, 2016). This scale was applied to both study groups.

*Family satisfaction*. It is defined as the positive or negative perception about the family functioning of the family system, which implies the levels of emotional closeness, the ability to adapt to changes, the quality of communication, and the way of solving problems. It was evaluated through the Family Satisfaction Scale (FSS). Made up of 10 Likert-type items with five response alternatives, whose valuation ranges from one (very dissatisfied) to five (extremely satisfied). Family satisfaction is defined as the level of satisfaction of the relationships they experience with other family members in terms of emotional closeness, communication, and flexibility to adapt to changes (Olson, Gorall & Tiesel, 2006). The original version has optimal levels of reliability (α = 0.92) and adequate evidence of internal structure (Olson, Gorall & Tiesel, 2006). The FSS has been studied in Peruvian university students, presenting evidence of one-dimensionality (CFI > 0.95, RMSEA < 0.08), as well as optimal reliability coefficients (α > 0.85) (Villarreal-Zegarra et al., 2017). This scale was applied to both study groups.

*Circumplex ratio scores*. Olson proposes using cohesion and flexibility ratios that identify the relationship between the balanced and unbalanced dimensions of cohesion and flexibility, respectively. When the ratio score is lower than the dimension being measured, the system will be more unbalanced. However, when the value of this ratio is higher, a more balanced system will be obtained (Olson, Gorall & Tiesel, 2006).

#### Covariates (first hypothesis)

Data was collected on sex (men, women), age (years), years of education (6 years or less, 7 to 11 years, and 12 years or more), whether they have children (yes/no), and civil status (married, divorced, widowed, cohabiting, separated, or single).

### Variables for the second hypothesis

#### Exposure (second hypothesis)

The exposure was family functioning, assessed by cohesion, flexibility, communication, and satisfaction. Also, Circumplex Ratio Scores were considered as an exposure variable. The Family Adaptability and Cohesion Evaluation Scale (FACES IV), Family Communication Scale (FCS), Family Satisfaction Scale (FSS), and Circumplex Ratio Scores were used to assess the exposures variables.

#### Outcome (second hypothesis)

The outcome was family compliance with quarantine. Data were collected from families assessed in 2020 on how they complied with quarantine (“strictly enforced”, “partially enforced or no quarantine compliance”). For this second hypothesis, we did not consider single-person families, *i.e*., those who live alone and therefore do not apply since they do not have other family members who may or may not comply with the quarantine.

#### Covariates (second hypothesis)

In addition to the covariates for the first hypothesis, information on the economic status of the families was added. First, if the families have received an economic bonus (universal family bonus) provided by the Peruvian Government to low-income families to help them cope with the pandemic (760 Peruvian soles or USD 212.8 tops). This bonus is given to the poorest families to help them meet their expenses during quarantine. Second, family income during the pandemic (“No income”, “Less than 280 dollars”, “Between 280 to 560 dollars”, “Between 560 to 1,120 dollars”, “Between 1,120 to 1,680 dollars”, “Between 1,680 to 2,800 dollars”, “Between 2,800 or more dollars”, “I do not know”), and every 3.57 Peruvian soles correspond to one U.S. dollar.

### Data analysis

Groups studied before (2019) and during (2020) the COVID-19 pandemic were described in terms of the abovementioned variables. For that description, we reported relative and simple frequencies.

#### Analysis for the first hypothesis

We fitted univariate linear regression models to evaluate the differences between family functioning before and during the COVID-19 pandemic in Peruvian families. In these models, the outcomes were each dimension of family functioning described above (one regression model per indicator), and the main predictor was to belong to either the 2019 or 2020 group (*i.e*., the baseline category was the 2019 group). Differences were estimated unadjusted and adjusted for sex, age, years of education, with/without children, and marital status. A sensitivity analysis was conducted in a sub-sample of heads of households with a similar distribution of marital status and number of children, aged 20–39, living in the same geographical region (Ancash) and with 12 or more years of education to see whether the association estimates for the first hypothesis vary.

#### Analysis for the second hypothesis

We fitted univariate Poisson regression models with robust variance for evaluating the association between family functioning and quarantine compliance (Barros & Hirakata, 2003). In these models, the outcome was quarantine compliance, and the exposure was the z scores of family functioning. Z-scores were used to facilitate comparisons since the FACES, FCS and FSS had different score ranges (7 to 35 and 10 to 50, respectively). Prevalence ratios were reported unadjusted and adjusted for sex, age, with/without children, marital status, access to governmental support (bonus), and family income. This analysis did not consider those living alone (*i.e*., outside the family system), had no information on family income or had missing data (see Flowchart).

All the statistical analyses are explained—in more detail—in Appendix A (including relevant codes) to ensure transparency and reproducibility. Statistical assumptions were explored. We reported 95% confidence intervals for all estimates and considered *p* values < 0.05 as statistically significant. We performed all data analyses with the STATA software, version 15 (StataCorp, 2017).

### Ethical considerations

The ethics committee of Universidad Católica los Ángeles de Chimbote (ULADECH-2020-11-12) has approved the study protocol. The research team was responsible for safeguarding the rights, safety, and wellbeing of the participants. Also, the data was anonymous and confidential. Participants accepted a virtual informed consent at the time of recruitment.

## Results

### Sociodemographic characteristics

**Figure 1:**
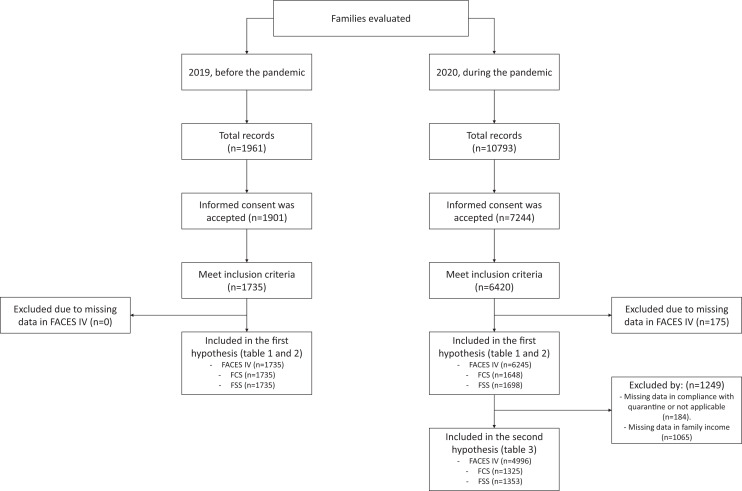
Flowchart of the study sample.

A total of 12,754 contacts were made, from which 9,145 accepted informed consent, 8,155 met the inclusion criteria and recorded full data on the variables of interest (Fig. 1). A total of 7,980 participants were included in the study, from where 6,245 (78.2%) were recruited during the COVID-19 pandemic in 2020. The majority of participants were female and single, in both years. Two-thirds of participants recruited before the pandemic had children (66%), while only 33% of participants recruited during the pandemic had children (Table 1). 51% of participants recruited during the pandemic reported that their families were in strict compliance with quarantine, and 33.3% had received state assistance for low income. We found optimal reliability values for several dimensions: disengaged (α = 0.79), chaotic (α = 0.83), balanced cohesion (α = 0.77) and balanced flexibility (α = 0.85), as well as family communication (α = 0.91) and family satisfaction (α = 0.91). However, it’s worth noting that the dimensions of enmeshed (α = 0.50) and rigid (α = 0.66) exhibited lower levels of reliability.

**Table 1:** Characteristic of the study participants.

		2019, before the pandemic (*n* = 1,735)		2020, during the pandemic (*n* = 6,245)	
		*n*	%	*n*	%
Sex	Women	948	54.6%	3,985	63.8%
	Men	787	45.4%	2,260	36.2%
Years of education	6 years or less	62	3.6%	–	–
	7 to 11 years old	718	41.4%	–	–
	12 years or more	955	55.0%	6,245	100.0%
With children	No	584	33.7%	4,372	70.0%
	Yes	1,151	66.3%	1,873	30.0%
Civil status	Married	399	23.0%	616	9.9%
	Divorced	46	2.7%	55	0.9%
	Widowed	34	2.0%	29	0.5%
	Cohabiting	509	29.3%	652	10.4%
	Separated	229	13.2%	163	2.6%
	Single	518	29.9%	4,730	75.7%
Age group	16 to 19	235	13.5%	1,440	23.1%
	20 to 29	610	35.2%	3,211	51.4%
	30 to 39	393	22.7%	998	16.0%
	40 to 49	243	14.0%	428	6.9%
	50 to 65	254	14.6%	168	2.7%
Family meets quarantine*	Strictly enforced	–	–	3,243	51.9%
	Partial enforced	–	–	2,731	43.7%
	Non-quarantine compliance	–	–	87	1.4%
	I live alone (not applicable)	–	–	172	2.8%
	Missing	–	–	12	0.2%
Receive a money bonus*	No	–	–	4,163	66.7%
	Yes	–	–	2,082	33.3%
Family income*	No income	–	–	1,559	19.5%
	Less than 280 dollars	–	–	1,842	23.1%
	280 to 560 dollars	–	–	999	12.5%
	560 to 1,120 dollars	–	–	491	6.2%
	1120 to 1,680 dollars	–	–	142	1.8%
	1,680 to 2,800 dollars	–	–	70	0.9%
	2,800 or more dollars	–	–	42	0.5%
	I do not know	–	–	1,100	13.8%

**Note:**

* This information is only available for participants evaluated in 2020. During the evaluation, the minimum wage in Peru was 260.00 U.S. dollar. *n* = number of participants. % = percentage.

#### Pandemic exposure on family functioning

The participants assessed during the pandemic had significant differences in family functioning compared to participants assessed before the pandemic (Table 2). Families assessed during the pandemic had higher scores on more functional dimensions such as Balanced Cohesion (β = 1.4) and Balanced Flexibility (β = 2.0) and have lower scores on dysfunctional dimensions such as Disengaged (β = −1.9) and Chaotic (β = −2.9). However, no family satisfaction or communication differences were identified in families before and during the pandemic. Participants assessed during the pandemic had higher scores on the Flexibility Ratio (β = 0.9) and Total Circumplex Ratio (β = 0.5), indicating greater capacity for change (flexibility) and balance between family cohesion and flexibility, respectively.

**Table 2:** Family functioning differences between independent groups evaluated before (2019) and during (2020) the COVID-19 pandemic (*N* = 7,980).

	2019, before the pandemic (*n* = 1,735)	2020, during the pandemic (*n* = 6,245)	Unadjusted mean difference (MD)	Adjusted mean difference* (MD*)
	Mean (SD)	Mean (SD)	MD (95% CI)	MD* (95% CI)
Balanced cohesion	25.8 (5.5)	26.9 (4.8)	**1.1 [0.8–1.4]**	**1.4 [1.0–1.7]**
Balanced flexibility	26.4 (5.6)	28.0 (5.1)	**1.5 [1.3–1.8]**	**2.0 [1.6–2.4]**
Disengaged	19.3 (6.3)	17.5 (5.8)	**−1.8 [−2.1 to −1.5]**	**−1.9 [−2.3 to −1.5]**
Enmeshed	21.1 (5.0)	20.8 (4.2)	−0.2 [−0.4 to 0.0]	0.0 [−0.3 to 0.3]
Rigid	22.2 (5.0)	21.9 (4.7)	−0.3 [−0.5 to 0.0]	0.0 [−0.4 to 0.3]
Chaotic	18.8 (6.8)	15.9 (5.9)	**−2.9 [−3.2 to −2.5]**	**−2.9 [−3.3 to −2.4]**
Family communication	38.9 (8.4)	38.5 (7.7)**	−0.4 [−1.0 to 0.1]	0.5 [−0.2 to 1.2]
Family satisfaction	39.2 (8.0)	38.8 (7.4)**	−0.4 [−0.9 to 0.1]	−0.1 [−0.7 to 0.6]
Cohesion ratio	2.0 (4.4)	2.0 (4.3)	−0.1 [−0.3 to 0.2]	0.1 [−0.2 to 0.4]
Flexibility ratio	1.6 (3.8)	2.1 (5.6)	**0.5 [0.2–0.8]**	**0.9 [0.5–1.2]**
Total circumplex ratio	1.8 (3.6)	2.0 (4.1)	0.2 [0.0–0.4]	**0.5 [0.2–0.7]**

**Notes:**

* Each mean difference was adjusted for sex, age, education, with/without children, and marital status.

** The number of participants for the family communication (*n* = 1,648) and family satisfaction (*n* = 1,698) were slightly different (see Fig. 1).

SD, standard deviation; CI, confidence interval. Values in bold are significant (*p* < 0.05). MD, mean difference.

Sensitivity analysis estimates (Appendix B) did not vary notably from original estimates (Table 2). The adjusted mean differences (AMD) between 2019 and 2020 participants remain equal in direction and equal, similar or partially similar in size for all dimensions: balanced cohesion (AMD: = 1.2, 95% CI [0.7–1.8] or 86% of the original AMD = 1.4 reported in Table 2), balanced flexibility (AMD = 2.0, 95% CI [1.3–2.6], which is the same AMD reported in Table 2), disengaged (AMD = −1.2 95% CI [−1.9 to −0.4] or 63% of the original AMD = 1.9 in Table 2) and chaotic (AMD = −2.2 95% CI [−3.0 to −1.5] or 76% of the original AMD = 2.9 in Table 2).

#### Family functioning and quarantine compliance

Strictly quarantine compliant families had higher scores on balanced dimensions such as Balanced Cohesion (aPR = 1.158) and Balanced Flexibility (aPR = 1.225) (Table 3). Families that strictly complied with quarantine had higher emotional bonding among their members as indicated by higher scores on the Enmeshed dimension (aPR = 1.043) and lower scores on the Disengaged dimension (aPR = 0.906), compared to those families that did not comply with quarantine or partially complied with quarantine. Families that strictly complied with quarantine also presented more rigid boundaries and rules, as higher scores on the Rigid dimension (aPR = 1.069) and lower scores on the Chaotic dimension (aPR = 0.893) were identified. These families also presented higher scores for family communication, family satisfaction, Cohesion Ratio, Flexibility Ratio, and Total Circumplex Ratio.

**Table 3:** Quarantine compliance and family functioning in people evaluated during the COVID-19 pandemic (*N* = 4,996, year 2020).

	Unadjusted prevalence ratio (PR)	Adjusted prevalence ratio* (aPR*)
	PR (95% CI)	aPR* (95% CI)
Balanced cohesion	**1.171 [1.136–1.206]**	**1.158 [1.124–1.193]**
Balanced flexibility	**1.253 [1.212–1.129]**	**1.225 [1.185–1.265]**
Disengaged	**0.899 [0.875–0.923]**	**0.906 [0.882–0.931]**
Enmeshed	**1.056 [1.030–1.083]**	**1.043 [1.018–1.069]**
Rigid	**1.077 [1.049–1.104]**	**1.069 [1.043–1.097]**
Chaotic	**0.884 [0.860–0.909]**	**0.893 [0.869–0.918]**
Family communication**	**1.192 [1.127–1.261]**	**1.171 [1.108–1.237]**
Family satisfaction**	**1.206 [1.138–1.279]**	**1.180 [1.113–1.250]**
Cohesion ratio	**1.013 [1.009–1.017]**	**1.011 [1.007–1.015]**
Flexibility ratio	**1.010 [1.007–1.013]**	**1.008 [1.005–1.011]**
Total circumplex ratio	**1.016 [1.013–1.020]**	**1.014 [1.010–1.018]**

**Notes:**

Quarantine compliance was analysed as a binary outcome (1 = strict compliance, 0 = partial or null compliance). Family functioning variables were treated as continuous. The values “I live alone”, “I do not know about my family income” or missing were not considered (see Fig. 1). CI, confidence interval. Values in bold are significant (*p* < 0.05). PR, Unadjusted prevalence ratio. aPR, Adjusted prevalence ratio.

* Estimates in each row come from an univariate robust-Poisson regression model adjusted for sex, age, with/without children, marital status, and family income.

** The number of participants for the family communication (*n* = 1,325) and family satisfaction (*n* = 1,353) were slightly different (see Fig. 1).

As an example, to facilitate interpretation, a person with 31.7 points on the balanced cohesion dimension (obtained from the mean 26.9 adding a standard deviation of 4.8) would be 15.8% more likely to strictly comply with quarantine (obtained from the adjusted prevalence ratio) compared to someone with 26.9 points (mean score). Therefore, for each additional standard deviation to the mean score obtained on a dimension, the probability of strictly complying with the quarantine increases or decreases as a function of the adjusted prevalence ratio.

## Discussion

### Main findings

We evaluated two hypotheses on the impact of COVID-19 on family functioning and the ability of balanced families to adapt to quarantine. For the first hypothesis, we found that (1) during the pandemic, families scored higher on cohesion and balanced flexibility, and (2) during the pandemic, families scored lower on disengaged and chaotic dimensions. An illustrative example that might clarify the results of the first hypothesis is that the circumstances of the pandemic forced families to increase the amount of time they spent together. As a result, they were forced to find more effective ways of adapting to the changed circumstances (quarantine). However, this adaptation wasn’t necessarily associated with increased satisfaction with family dynamics or improved communication. Rather, it indicated a strengthening of the emotional bond between family members and an increased capacity to respond skilfully to the evolving demands of the environment.

Regarding the second hypothesis, we confirmed that (3) families with balanced cohesion and flexibility were associated with greater compliance with quarantine restrictions; (4) disengaged and chaotic families were more likely to partially comply or not comply with the quarantine; and (5) family communication and satisfaction were also associated with greater quarantine compliance.

### Comparison with other studies

#### First hypothesis: family functioning changed before and during the COVID-19 pandemic

The COVID-19 pandemic crisis triggered new challenges for families to cover their basic needs, consequently impacting family relationships and functioning, now adapted to cope with these challenges. In China, rural migrant families suffered a high impact on their fragile economies and working lives (Tang & Li, 2021). This obligated them to reorganise the roles and activities at home, allowing families to respect the new lockdown restrictions while covering all the basic and emerging needs and medical treatment when possible. This is also a fair description of what the Peruvian families had to do during the pandemic restrictions. With variations in human capital, family burdens, economic support from the Government, access to remote work and education, Peruvian families learned new ways to ensure their basic needs. This learning occurred in a relatively short period, forced by the unexpected and never-lived circumstances, and, as in any critical scenario that is external and out of the family control (*e.g*., COVID-19 pandemic), it demanded the cooperation and positive predisposition of all members. In such a circumstance, people are more flexible to assume new roles and negotiate or accept new rules at home. For example, to take care of someone who got sick or partially relieve their leadership if the ill person is one of the family heads. When the family was economically fragile, having COVID-19 at home represented one of the highest stressors, inducing parents to be reactive, inconsistent and even aggressive, amplifying, in turn, the stress among parents and children (Daks, Peltz & Rogge, 2020). However, parent flexibility was protective against family discord and was linked to more constructive parenting strategies (*e.g*., democratic/autonomy-supportive) (Daks, Peltz & Rogge, 2020). Thus, balanced flexibility is an alternative that some parents should have identified as the best to take during the crisis, finding in their children the receptive attitude needed to complete the change successfully.

Apart from the challenges explained above, the pandemic appears to have forced family members to spend more time in the same space—tiny in many cases—increasing the stress at home, which is already high (Olson, Waldvogel & Schlieff, 2019). However, as the theory predicts, families usually find a way to cope with it (third assumption of the Circumplex Model). In Australia, despite the critical problems, some families found benefits from the lockdown, such as strengthening relationships, finding new hobbies, and developing appreciation, gratitude and tolerance (Evans et al., 2020). Using their own words: ‘Spending more time together has strengthened bonds’. More than 60% of families were functionally connected in Portugal, while 94% reported a midrange or balanced functioning during the pandemic (Fernandes et al., 2021). Considering this evidence and ours from Peru, it seems like the transit towards a balanced functioning during the pandemic can be culturally independent. However, there may also be individual factors that influence the balanced response of family relationships, such as the resilience of family members (Guazzelli Williamson et al., 2022).

It is worth reflecting on how family functioning scores changed during the pandemic. Families assessed during the pandemic had lower scores on the unbalanced, disjointed and chaotic dimensions and higher scores on the family cohesion and flexibility dimensions, compared to families before the pandemic. This has at least two probable explanations. First, in many Latin American contexts having the strongest family ties and following parents’ leadership in a vertical relationship is culturally appreciated (Puentes Gómez, 2014). Extended families are not atypical (*i.e*., grandparents, parents, children, uncles and aunts, councils, all living in the same house), expressing how enmeshed relationships continue across generations. In a culture where togetherness is a synonym of family, the enmeshed level can be unbalanced but not necessarily dysfunctional (Olson, Waldvogel & Schlieff, 2019). Moreover, if extreme family cohesion works all right in a crisis context like the pandemic, families do not have a reason to change. This leads us to the second reason: unbalanced families in the direction of more cohesion and less flexibility can be functional enough in a crisis such as an epidemic/pandemic. In Peruvian families, rigid structures of roles and rules can help to organise the limited resources available to survive long-term crises, always it is accompanied by responsibility and cooperation.

This more balanced performance observed between the two measurements, presumably achieved in the context of the COVID-19 pandemic, may be temporary. We believe new cohesion levels can stay for a while, like some new rules, but some roles will return to normal. For example, when an adolescent moves into young adulthood, the rules of the family system are reduced because independence is gained. In critical situations such as one or both parents being ill due to COVID-19 pandemic, some adolescents had to assume some adult responsibilities earlier than expected, gaining some independence in due course. When parents recover their health, the adult responsibilities come back to them, but the independence gained by the adolescent will stay (Siguenza, Buñay & Guamán-Arias, 2017). However, we would not expect the families to return to the same functioning before the pandemic.

Our findings confirmed that this change in cohesion and flexibility levels during the pandemic did not compromise families’ satisfaction levels seen before the pandemic. However, these results must be read with caution. In a study performed in North Carolina (USA), Hussong et al. (2022) children reported decreased family satisfaction but no changes in what parents reported. Williamson (2020) independently reported no changes in relationship satisfaction among adults in the US. In Peru, we found no change in family satisfaction reported by adults, which is relatively consistent with previous studies. Nevertheless, the children’s perspective can be different from the adults’; thus, more research is needed to conclude.

Surprisingly, no improvements in family communication were needed to help the adaptation process. We presume that social media and streaming services could play a role here (Liu, Zhu & Xia, 2021; Zhou & Yu, 2021) by managing to supplant family members’ need for socialising and limiting the time for in-person interactions between family members. On the other hand, complying with new public restrictions to reduce contagion (*e.g*., use of masks, social distancing, not leaving home) has induced families to develop clearer boundaries at home (*e.g*., changing some roles and rules). This could have affected family satisfaction, but there was no significant change, as we confirmed. Nevertheless, it seems like the communication level at baseline (before the pandemic started) was enough to ensure family adaptation.

One potential source of bias in our study was the difference in characteristics between participants from 2019 and 2020. We acknowledge that variations in the family role and composition may affect the perception and assessment of family dynamics by respondents. The sensitivity analysis performed to address this issue showed a small bias that did not compromise the study conclusions. For all family functioning dimensions, the difference between 2019 and 2020 always had the same direction and, in the most conservative scenario, >60% of the magnitude originally detected.

#### Second hypothesis: functional families are more likely to comply with strict quarantine

The restriction measures meant that families had to quickly adapt to new habits to prevent the spread of the virus, such as mask use, quarantine, teleworking, and avoiding crowded places. The changes in habits, the uncertainty experienced during the first year of the pandemic, and the fear of becoming infected with the virus have generated a considerable increase in family stress, which was greater in families where parents were more inflexible or unbalanced families (Chung et al., 2023). Unbalanced families, in addition to presenting problems for their members to relate satisfactorily with each other, during the pandemic have seen reduced independence of its members because they had to remain inside their homes daily and could not go out to study, socialise or work. A mixed study conducted in the United States found that university students who returned to live with their families during the quarantine had a more negative view of their relationship with their parents, decreased their autonomy, and showed less positive coping (Hall & Zygmunt, 2021). Although in Peru, the rate of students who become independent to go to college is not as high as in the United States, Peruvian youth gain much independence during the college stage as they manage to establish their schedules and routines. In Peru, a phenomenon similar to that experienced by university students in the United States could have occurred, where the fact of being deprived of their primary socialisation (the university people), being in a context of high uncertainty such as a pandemic, and having reduced social support, caused family members not to respect the quarantine as strictly.

Balanced families are better able to adapt and cope with a crisis, such as the illness of one of their members or having to comply strictly with a quarantine. A study of mothers with type 2 diabetes found that families with better family communication strategies and those who could redistribute their roles achieved better diabetes self-management (Fisher et al., 2020). This is because they reduce risk behaviours by better controlling diet, adopting healthy behaviours, and generating spaces for conversation about lifestyle changes (Fisher et al., 2020). Translating this evidence to the context of COVID-19 pandemic, balanced families may be able to organise themselves in such a way as to reduce risk behaviours such as leaving the house, increasing compliance with quarantine.

### Public health implications

The combined effect of reducing unbalanced dimensions and increasing balanced dimensions represents a natural advantage during times of crisis, such as a pandemic (Olson, 2000). Family functioning is crucial for compliance with quarantine measures, and a balanced functioning can be previously strengthened. For example, programs for promoting a balanced family functioning can be given in the schools through development activities already set for parents (in Peru, the so-called “School for Parents”). This type of family-focused intervention can be given in the long-term (increasing chances of effective change) and has proven to be helpful to improve the control of other problems, such as programs for childhood obesity control (Silver & Cronin, 2019) and asthma control in children (Weinstein & Faust, 1997). Families with more balanced functioning, or even with the knowledge of its advantages, can be more prepared to cope with this type of crisis and cooperate more with restriction measures such as quarantines, which directly slow down the spread of contagion.

### Strengths and limitations

This is the first study of its kind performed in families from LMIC, providing strong evidence for both the particular case of the COVID-19 pandemic and the more general Circumplex Model assumptions. However, our study has some limitations. First, other family variables may have affected the associations assessed, such as family resilience, family composition, wellbeing, belief system or parenting stress (Prime, Wade & Browne, 2020). Finally, our study design does not allow us to reach causal conclusions; thus, all our findings, interpretations and discussions about them must be understood as related to association evidence only.

## Conclusions

Family functioning changed after the pandemic initiation, showing higher balanced functioning (cohesion and flexibility) and lower unbalanced levels (disengaged and chaotic). During the pandemic, balanced families had more capacity to comply with quarantine than unbalanced families. This new evidence enlightens the family systems theory (*i.e*., Circumplex Model) while informing future interventions for improving compliance with quarantine measures in the context of social confinement.

## Supplemental Information

10.7717/peerj.16269/supp-1 Supplemental Information 1 Appendices.

## Additional Information and Declarations

### Data Availability

The following information was supplied regarding data availability: The data is available at Figshare: Villarreal-Zegarra, David; Bazo Alvarez, Juan Carlos (2023). Differences on family functioning before and during the COVID-19 pandemic: an observational study in Peruvian families. figshare. Dataset. https://doi.org/10.6084/m9.figshare.22110599.v2.
