# Supplementary material for: Differences in family functioning before and during the COVID-19 pandemic: an observational study in Peruvian families
Source: PeerJ. 2023 Dec 8;11:e16269. doi: 10.7717/peerj.16269 (PMC10712306; doi:10.7717/peerj.16269)
Supplement: Supplemental Information 1 [file peerj-11-16269-s001.docx]

## **APPENDICES**

### **A****ppendix A. Do in STATA.**

**************

*CODEBOOK

**************

Age= Variable number of age

Sex= Categorical variable of sex (Women; Men)

Education= Categorical variable of

Group_children= Categorical variable if have a children (No; Yes)

Civilstatus = Categorical variable of Civil status (Married; Divorced; Widowed; Cohabiting; Separated; Single)

Agegroup= Categorical variable of Years of education (6 years or less; 7 to 11 years old; 12 years or more)

Meetsquarantine = Categorical variable if the family meets quarantine (Strictly enforced; Partial enforced; non-quarantine compliance; I live alone (not applicable); Missing)

Receivedbonusquarantine= Categorical variable if receive a money bonus (No; Yes)

IncomeDuring= Categorical variable of family income (No income; Less than 280 dollars; 280 to 560 dollars; 560 to 1120 dollars; 1120 to 1680 dollars; 1680 to 2800 dollars; 2800 or more dollars; I do not know)

FACESCohesionbal = Numerical variable of the balanced cohesion dimension of FACES IV.

FACESFlexibilityBal = Numerical variable of the balanced flexibility dimension of FACES IV.

FACESDisengaged = Numerical variable of the disengaged dimension of FACES IV.

FACESEnmeshed = Numerical variable of the enmeshed dimension of FACES IV.

FACESRigid = Numerical variable of the rigid dimension of FACES IV.

FACESChaotic = Numerical variable of the chaotic dimension of FACES IV.

FCS = Numerical variable of the Family communication scale

FSS = Numerical variable of the Family Satisfaction scale

CohesionRatio = Numerical variable of Cohesion Ratio

FlexibilityRatio = Numerical variable of Flexibility Ratio

TotalCircumplexRatio = Numerical variable of Total Circumplex Ratio

generate FACESCohesionbalSD = (FACESCohesionbal - 26.95516)/4.80511

generate FACESFlexibilityBalSD = (FACESFlexibilityBal - 28.02822)/5.122386

generate FACESDisengagedSD = (FACESDisengaged - 17.43595)/5.734159

generate FACESEnmeshedSD = (FACESEnmeshed - 20.93555)/4.269388

generate FACESRigidSD = (FACESRigid - 21.92134)/4.731427

generate FACESChaoticSD = (FACESChaotic - 15.97998)/5.941321

generate FCSSD = (FCS - 38.5766)/7.765029

generate FSSSD = (FSS - 38.93126)/7.397155

**************

*TABLE 1

**************

tabstat Age, statistics( mean sd ) by(Year)

tab Sex Year, m

tab education Year, m

tab Group_children Year, m

tab Civilstatus Year, m

tab Agegroup Year, m

*Only for families of 2020

tab Meetsquarantine Year, m

tab Receivedbonusquarantine Year, m

tab IncomeDuring Year, m

**************

*TABLE 2

**************

tabstat FACESCohesionbal FACESFlexibilityBal FACESDisengaged FACESEnmeshed FACESRigid FACESChaotic FCS FSS, statistics( min max mean mean sd ) by(Year)

*Raw model

regress FACESCohesionbal i.b2.Year, cformat(%9.1f) pformat(%5.3f) sformat(%8.3f)

regress FACESFlexibilityBal i.b2.Year, cformat(%9.1f) pformat(%5.3f) sformat(%8.3f)

regress FACESDisengaged i.b2.Year, cformat(%9.1f) pformat(%5.3f) sformat(%8.3f)

regress FACESEnmeshed i.b2.Year, cformat(%9.1f) pformat(%5.3f) sformat(%8.3f)

regress FACESRigid i.b2.Year, cformat(%9.1f) pformat(%5.3f) sformat(%8.3f)

regress FACESChaotic i.b2.Year, cformat(%9.1f) pformat(%5.3f) sformat(%8.3f)

regress FCS i.b2.Year, cformat(%9.1f) pformat(%5.3f) sformat(%8.3f)

regress FSS i.b2.Year, cformat(%9.1f) pformat(%5.3f) sformat(%8.3f)

regress CohesionRatio i.b2.Year, cformat(%9.1f) pformat(%5.3f) sformat(%8.3f)

regress FlexibilityRatio i.b2.Year, cformat(%9.1f) pformat(%5.3f) sformat(%8.3f)

regress TotalCircumplexRatio i.b2.Year, cformat(%9.1f) pformat(%5.3f) sformat(%8.3f)

*Ajusted model

regress FACESCohesionbal i.b2.Year Age i.education i.Civilstatus i.Sex i.Group_children, cformat(%9.1f) pformat(%5.3f) sformat(%8.3f)

regress FACESFlexibilityBal i.b2.Year Age i.education i.Civilstatus i.Sex i.Group_children, cformat(%9.1f) pformat(%5.3f) sformat(%8.3f)

regress FACESDisengaged i.b2.Year Age i.education i.Civilstatus i.Sex i.Group_children, cformat(%9.1f) pformat(%5.3f) sformat(%8.3f)

regress FACESEnmeshed i.b2.Year Age i.education i.Civilstatus i.Sex i.Group_children, cformat(%9.1f) pformat(%5.3f) sformat(%8.3f)

regress FACESRigid i.b2.Year Age i.education i.Civilstatus i.Sex i.Group_children, cformat(%9.1f) pformat(%5.3f) sformat(%8.3f)

regress FACESChaotic i.b2.Year Age i.education i.Civilstatus i.Sex i.Group_children, cformat(%9.1f) pformat(%5.3f) sformat(%8.3f)

regress FCS i.b2.Year Age i.education i.Civilstatus i.Sex i.Group_children, cformat(%9.1f) pformat(%5.3f) sformat(%8.3f)

regress FSS i.b2.Year Age i.education i.Civilstatus i.Sex i.Group_children, cformat(%9.1f) pformat(%5.3f) sformat(%8.3f)

regress CohesionRatio i.b2.Year Age i.education i.Civilstatus i.Sex i.Group_children, cformat(%9.1f) pformat(%5.3f) sformat(%8.3f)

regress FlexibilityRatio i.b2.Year Age i.education i.Civilstatus i.Sex i.Group_children, cformat(%9.1f) pformat(%5.3f) sformat(%8.3f)

regress TotalCircumplexRatio i.b2.Year Age i.education i.Civilstatus i.Sex i.Group_children, cformat(%9.1f) pformat(%5.3f) sformat(%8.3f)

**************

*TABLE 3

**************

*Raw model

poisson Meetsquarantine2 FACESCohesionbalSD if IncomeDuring!=7&Meetsquarantine !=0, cformat(%9.3f) pformat(%5.3f) sformat(%8.3f) irr vce(robust)

poisson Meetsquarantine2 FACESFlexibilityBalSD if IncomeDuring!=7&Meetsquarantine !=0, cformat(%9.3f) pformat(%5.3f) sformat(%8.3f) irr vce(robust)

poisson Meetsquarantine2 FACESDisengagedSD if IncomeDuring!=7&Meetsquarantine !=0, cformat(%9.3f) pformat(%5.3f) sformat(%8.3f) irr vce(robust)

poisson Meetsquarantine2 FACESEnmeshedSD if IncomeDuring!=7&Meetsquarantine !=0, cformat(%9.3f) pformat(%5.3f) sformat(%8.3f) irr vce(robust)

poisson Meetsquarantine2 FACESRigidSD if IncomeDuring!=7&Meetsquarantine !=0, cformat(%9.3f) pformat(%5.3f) sformat(%8.3f) irr vce(robust)

poisson Meetsquarantine2 FACESChaoticSD if IncomeDuring!=7&Meetsquarantine !=0, cformat(%9.3f) pformat(%5.3f) sformat(%8.3f) irr vce(robust)

poisson Meetsquarantine2 FCSSD if IncomeDuring!=7&Meetsquarantine !=0, cformat(%9.3f) pformat(%5.3f) sformat(%8.3f) irr vce(robust)

poisson Meetsquarantine2 FSSSD if IncomeDuring!=7&Meetsquarantine !=0, cformat(%9.3f) pformat(%5.3f) sformat(%8.3f) irr vce(robust)

poisson Meetsquarantine2 CohesionRatio if IncomeDuring!=7&Meetsquarantine !=0, cformat(%9.3f) pformat(%5.3f) sformat(%8.3f) irr vce(robust)

poisson Meetsquarantine2 FlexibilityRatio if IncomeDuring!=7&Meetsquarantine !=0, cformat(%9.3f) pformat(%5.3f) sformat(%8.3f) irr vce(robust)

poisson Meetsquarantine2 TotalCircumplexRatio if IncomeDuring!=7&Meetsquarantine !=0, cformat(%9.3f) pformat(%5.3f) sformat(%8.3f) irr vce(robust)

*Ajusted model

poisson Meetsquarantine2 FACESCohesionbalSD Age i.Civilstatus i.Sex i.Bono i.Group_children IncomeDuring if IncomeDuring!=7&Meetsquarantine !=0, cformat(%9.3f) pformat(%5.3f) sformat(%8.3f) irr vce(robust)

poisson Meetsquarantine2 FACESFlexibilityBalSD Age i.Civilstatus i.Sex i.Bono i.Group_children IncomeDuring if IncomeDuring!=7&Meetsquarantine !=0, cformat(%9.3f) pformat(%5.3f) sformat(%8.3f) irr vce(robust)

poisson Meetsquarantine2 FACESDisengagedSD Age i.Civilstatus i.Sex i.Bono i.Group_children IncomeDuring if IncomeDuring!=7&Meetsquarantine !=0, cformat(%9.3f) pformat(%5.3f) sformat(%8.3f) irr vce(robust)

poisson Meetsquarantine2 FACESEnmeshedSD Age i.Civilstatus i.Sex i.Bono i.Group_children IncomeDuring if IncomeDuring!=7&Meetsquarantine !=0, cformat(%9.3f) pformat(%5.3f) sformat(%8.3f) irr vce(robust)

poisson Meetsquarantine2 FACESRigidSD Age i.Civilstatus i.Sex i.Bono i.Group_children IncomeDuring if IncomeDuring!=7&Meetsquarantine !=0, cformat(%9.3f) pformat(%5.3f) sformat(%8.3f) irr vce(robust)

poisson Meetsquarantine2 FACESChaoticSD Age i.Civilstatus i.Sex i.Bono i.Group_children IncomeDuring if IncomeDuring!=7&Meetsquarantine !=0, cformat(%9.3f) pformat(%5.3f) sformat(%8.3f) irr vce(robust)

poisson Meetsquarantine2 FCSSD Age i.Civilstatus i.Sex i.Bono i.Group_children IncomeDuring if IncomeDuring!=7&Meetsquarantine !=0, cformat(%9.3f) pformat(%5.3f) sformat(%8.3f) irr vce(robust)

poisson Meetsquarantine2 FSSSD Age i.Civilstatus i.Sex i.Bono i.Group_children IncomeDuring if IncomeDuring!=7&Meetsquarantine !=0, cformat(%9.3f) pformat(%5.3f) sformat(%8.3f) irr vce(robust)

poisson Meetsquarantine2 CohesionRatio Age i.Civilstatus i.Sex i.Bono i.Group_children IncomeDuring if IncomeDuring!=7&Meetsquarantine !=0, cformat(%9.3f) pformat(%5.3f) sformat(%8.3f) irr vce(robust)

poisson Meetsquarantine2 FlexibilityRatio Age i.Civilstatus i.Sex i.Bono i.Group_children IncomeDuring if IncomeDuring!=7&Meetsquarantine !=0, cformat(%9.3f) pformat(%5.3f) sformat(%8.3f) irr vce(robust)

poisson Meetsquarantine2 TotalCircumplexRatio Age i.Civilstatus i.Sex i.Bono i.Group_children IncomeDuring if IncomeDuring!=7&Meetsquarantine !=0, cformat(%9.3f) pformat(%5.3f) sformat(%8.3f) irr vce(robust)

**************

*Appendix C. Sensitivity analysis

**************

tabstat FACESCohesionbal FACESFlexibilityBal FACESDisengaged FACESEnmeshed FACESRigid FACESChaotic FCS FSS CohesionRatio FlexibilityRatio TotalCircumplexRatio if education==2, statistics( mean sd ) by(Year)

*Raw model

regress FACESCohesionbal i.b2.Year if education==2 , cformat(%9.1f) pformat(%5.3f) sformat(%8.3f)

regress FACESFlexibilityBal i.b2.Year if education==2 , cformat(%9.1f) pformat(%5.3f) sformat(%8.3f)

regress FACESDisengaged i.b2.Year if education==2 , cformat(%9.1f) pformat(%5.3f) sformat(%8.3f)

regress FACESEnmeshed i.b2.Year if education==2 , cformat(%9.1f) pformat(%5.3f) sformat(%8.3f)

regress FACESRigid i.b2.Year if education==2 , cformat(%9.1f) pformat(%5.3f) sformat(%8.3f)

regress FACESChaotic i.b2.Year if education==2 , cformat(%9.1f) pformat(%5.3f) sformat(%8.3f)

regress FCS i.b2.Year if education==2 , cformat(%9.1f) pformat(%5.3f) sformat(%8.3f)

regress FSS i.b2.Year if education==2 , cformat(%9.1f) pformat(%5.3f) sformat(%8.3f)

regress CohesionRatio i.b2.Year if education==2 , cformat(%9.1f) pformat(%5.3f) sformat(%8.3f)

regress FlexibilityRatio i.b2.Year if education==2 , cformat(%9.1f) pformat(%5.3f) sformat(%8.3f)

regress TotalCircumplexRatio i.b2.Year if education==2 , cformat(%9.1f) pformat(%5.3f) sformat(%8.3f)

*Ajusted model

regress FACESCohesionbal i.b2.Year Age i.Civilstatus i.Sex i.Group_children if education==2 , cformat(%9.1f) pformat(%5.3f) sformat(%8.3f)

regress FACESFlexibilityBal i.b2.Year Age i.Civilstatus i.Sex i.Group_children if education==2 , cformat(%9.1f) pformat(%5.3f) sformat(%8.3f)

regress FACESDisengaged i.b2.Year Age i.Civilstatus i.Sex i.Group_children if education==2 , cformat(%9.1f) pformat(%5.3f) sformat(%8.3f)

regress FACESEnmeshed i.b2.Year Age i.Civilstatus i.Sex i.Group_children if education==2 , cformat(%9.1f) pformat(%5.3f) sformat(%8.3f)

regress FACESRigid i.b2.Year Age i.Civilstatus i.Sex i.Group_children if education==2 , cformat(%9.1f) pformat(%5.3f) sformat(%8.3f)

regress FACESChaotic i.b2.Year Age i.Civilstatus i.Sex i.Group_children if education==2 , cformat(%9.1f) pformat(%5.3f) sformat(%8.3f)

regress FCS i.b2.Year Age i.Civilstatus i.Sex i.Group_children if education==2 , cformat(%9.1f) pformat(%5.3f) sformat(%8.3f)

regress FSS i.b2.Year Age i.Civilstatus i.Sex i.Group_children if education==2 , cformat(%9.1f) pformat(%5.3f) sformat(%8.3f)

regress CohesionRatio i.b2.Year Age i.Civilstatus i.Sex i.Group_children if education==2 , cformat(%9.1f) pformat(%5.3f) sformat(%8.3f)

regress FlexibilityRatio i.b2.Year Age i.Civilstatus i.Sex i.Group_children if education==2 , cformat(%9.1f) pformat(%5.3f) sformat(%8.3f)

regress TotalCircumplexRatio i.b2.Year Age i.Civilstatus i.Sex i.Group_children if education==2 , cformat(%9.1f) pformat(%5.3f) sformat(%8.3f)

### **Appendix B. Sensitivity analysis on the participants with higher education only, with 20 to 39 years, and that living in Ancash.**

Family functioning differences between independent groups evaluated before (2019) and during (2020) the COVID-19 pandemic for participants with 12 years of education or more, with 20 to 39 years, and that living in Ancash (n=1,357).

|  | 2019, before the pandemic (n=628) | 2020, during the pandemic (n=729) | Unadjusted mean difference (MD) | Adjusted mean difference* (MD*) |
| --- | --- | --- | --- | --- |
|  | mean (sd) | mean (sd) | MD [95% CI] | MD* [95% CI] |
| Balanced Cohesion | 25.7 (5.1) | 26.8 (4.8) | **1.1 [0.5 to 1.6]** | **1.2 [0.7 to 1.8]** |
| Balanced Flexibility | 26.4 (5.4) | 28.1 (5.2) | **1.7 [1.2 to 2.3]** | **2.0 [1.3 to 2.6]** |
| Disengaged | 19 (6.2) | 17.6 (5.8) | **-1.4 [-2.0 to -0.7]** | **-1.2 [-1.9 to -0.4]** |
| Enmeshed | 21.1 (4.8) | 21 (4.5) | -0.1 [-0.6 to 0.4] | 0.2 [-0.3 to 0.8] |
| Rigid | 22.1 (4.9) | 22.2 (4.8) | 0.1 [-0.4 to 0.6] | 0.4 [-0.2 to 0.9] |
| Chaotic | 18.7 (6.7) | 16 (6) | **-2.7 [-3.4 to -2.1]** | **-2.2 [-3.0 to -1.5]** |
| Family communication | 38.6 (8.7) | 38.5 (8) | -0.1 [-1.5 to 1.2] | 0.4 [-1.0 to 1.9] |
| Family Satisfaction | 39.3 (8.1) | 39.1 (7.7) | -0.2 [-1.4 to 1.0] | 0.3 [-1.0 to 1.6] |
| Cohesion Ratio | 1.9 (3.7) | 1.7 (2.7) | -0.2 [-0.5 to 0.2] | -0.3 [-0.7 to 0.1] |
| Flexibility Ratio | 1.5 (3) | 1.7 (2.8) | 0.2 [-0.1 to 0.5] | 0.2 [-0.2 to 0.5] |
| Total Circumplex Ratio | 1.7 (3.1) | 1.7 (2.3) | -0.0 [-0.3 to 0.3] | -0.1 [-0.4 to 0.3] |

(*) Each mean difference was adjusted for sex, age, with/without children, and marital status.

** The number of participants for the family communication (n=836) and family satisfaction (n=866) were different (see Figure 1).

sd = standard deviation; CI = confidence interval. **Values in bold are significant (p<0.05)**.

*Interpretation:* Because the population evaluated before the pandemic and the population evaluated during the pandemic had different levels of education, it was decided to perform a sensitivity analysis. That is, to select participants from both groups and redo the analyses to identify whether the results remained stable. This analysis (Appendix B) showed that the results did not vary, so the results are assumed to be stable between the two groups.
